# Trends in the Application of Citizen Science in Waterbird Conservation: A Bibliometric Analysis

**DOI:** 10.3390/ani15030368

**Published:** 2025-01-27

**Authors:** Ruilin Wang, Keming Ma

**Affiliations:** 1 State Key Laboratory for Ecological Security of Regions and Cities, Research Center for Eco-Environmental Sciences, Chinese Academy of Sciences, Beijing 100085, China; wangruilin18@mails.ucas.ac.cn; 2 College of Resources and Environment, University of Chinese Academy of Sciences, Beijing 100049, China

**Keywords:** waterbird conservation, citizen science, trends, bibliometric

## Abstract

Waterbirds serve as indicator species for the quality and health of wetland ecosystems, and their conservation is of critical significance for global biodiversity. Citizen science has gradually emerged in recent years, playing an increasingly positive role in scientific research, particularly in ornithological studies. However, a systematic description of the application of citizen science data in waterbird conservation remains lacking. Bibliometrics is an effective method for analyzing the development of scientific disciplines, exploring trends, and examining thematic evolution. This paper utilizes bibliometric analysis of citation data from the Web of Science database, covering the period from 1970 to September 2024. The analysis reveals that this research field has undergone three distinct developmental phases, with a significant increase in annual publication volume during the third phase. Research focus has shifted from specific species and types of waterbirds to key hotspots and ecological phenomena. Future research hotspots are expected to include migratory birds, China, citizen science, and biodiversity. Influential papers within the field emphasize that the primary focus of waterbird conservation is habitat protection and the construction of habitat networks. As the discipline has developed, there is growing recognition that increasing public awareness of waterbird conservation, starting with student education, plays a crucial role in the accumulation of citizen science data and the advancement of waterbird conservation efforts.

## Simple Summary

The conservation of waterbirds is of significant importance to global biodiversity, and citizen science, as an emerging form of data accumulation in recent years, plays a key role in waterbird conservation research. This study conducts a bibliometric analysis of the application of citizen science in waterbird conservation, exploring the development process of this research field and gaining insights into the current state of research. Initially, citizen science-based waterbird conservation studies focused on specific species, later shifting attention to active areas of waterbird habitats, and in recent years, there has been an increasing focus on raising public awareness regarding waterbird conservation. This shift not only enhances the effectiveness of waterbird conservation efforts but also provides direction for future research trends. Furthermore, raising public awareness about waterbird conservation can facilitate data accumulation, thereby fostering a positive feedback loop for citizen science-based research in waterbird conservation.

## 1. Introduction

Waterbirds, as representative species highly dependent on coastal wetlands, are an important component of global biodiversity [1]. Their diversity and species abundance reflect the conservation and management status of wetlands, and their high environmental sensitivity makes them indicator species for the quality and health of wetland ecosystems [2,3,4]. Global wetland loss and degradation, extreme climate events, overhunting, and biological invasions are all contributing factors to the decline in waterbird populations, which in turn have negative impacts on ecosystems [5,6,7]. According to reports by Wetlands International, approximately 23% of global waterbird populations are in decline, and some species have not been recorded in the wild in recent years [8,9]. Given this alarming situation, waterbird conservation is attracting increasing attention.

Various methods are employed in waterbird research, such as field surveys, banding records, GPS tracking, and isotopic tagging [10,11,12]. Although these methods are effective, they often require a high level of expertise. Citizen science data, collected over large spatial and temporal scales, offer advantages such as low entry barriers, extensive spatial coverage, and long-term data collection [13,14]. In recent years, citizen science has been widely applied in scientific research. The concept of “citizen science” was first introduced by researchers at Cornell University’s Lab of Ornithology and has since evolved from basic data collection to data analysis, model applications based on data, and further research based on data [15]. The data are primarily sourced from well-known platforms such as GBIF (Global Biodiversity Information Facility), BBS (North American Breeding Bird Survey), and Movebank [16]. These data not only include observational data but also group-provided survey and tracking data [17]. Increasing research has confirmed that citizen science data can be effectively applied in fields such as ornithological studies and the formulation of conservation policies [18,19]. However, no bibliometric studies have yet been conducted to assess the application of citizen science data in waterbird conservation.

Bibliometrics, which can combine theories such as the theory of scientific revolutions, structural hole theory, and foraging theory, provides a visual means of exploring the development of a discipline [20]. It allows for the rapid filtering of irrelevant information from large volumes of literature, delineating the developmental trajectory of the discipline, identifying key evolutionary nodes, and efficiently highlighting critical information [21]. This study employs the bibliometric software CiteSpace 6.4.R1 to analyze all literature indexed in the Web of Science core database from 1970 to 2024 on the application of citizen science in waterbird conservation [20]. This analysis aims to elucidate the developmental context of the field, identify current research hotspots, and pinpoint key milestones in the evolution of the discipline. The findings provide valuable data references and insights for waterfowl conservation research, thereby facilitating the deeper advancement of this field.

## 2. Materials and Methods

### 2.1. Literature Retrieval Strategy

The data source for this study was the Web of Science Core Collection SCI Expanded database. Based on bibliometric literature retrieval theory, the search strategy was formulated as follows: TS = ((waterbird* OR waterfowl*) AND conservation AND (“citizen science” OR GBIF OR BBS OR Movebank)) [22]. The document types were limited to “article” “review article” and “book chapters”. Duplicate records from the search results were removed. Ultimately, a total of 2604 articles were retrieved from the Web of Science Core Collection, spanning from 1970 to September 2024.

### 2.2. Bibliometric Analysis

Bibliometric analysis generally includes five steps: research design, data collection, data analysis, data visualization, and interpretation [23]. In the research design phase, the study theme was identified, and relevant search keywords, search strings, and data sources were determined. During the data collection phase, a total of 3708 documents were retrieved through literature searches. Using the Web of Science (WOS) literature type filter, the relevant document types for this study were selected, resulting in a final dataset of 2604 publications, spanning from 1970 to September 2024, which were exported in plain text format. In the data analysis phase, these plain text records were imported into bibliometric software for computation. An overview of the publication records utilized in this study is provided in Table 1. Significant results were used to construct citation networks and other visualizations for graphical representation.

The commonly utilized methodologies in bibliometric analysis encompass analysis of research areas, analysis of research hotspots, evolution of research themes, and analysis of thematic clusters [21].

Each publication in the Web of Science (WOS) database is classified into a specific research area. Clustering these research areas provides a comprehensive understanding of the evolution of research content and helps identify key focus areas [24].

Analyzing keyword bursts in academic publications helps capture research hotspots and their evolutionary trends at different stages of disciplinary development, providing a clearer understanding of the field’s trajectory [21].

The knowledge base of a research field is constructed from co-cited references, while the corresponding citations (i.e., original data) represent the research frontiers of different developmental stage citation clustering networks generated from the original data can reveal detailed research themes [21]. By examining the connections between themes and clusters, the evolution of research themes within the field can be traced [20].

In CiteSpace, the clustering results are evaluated using two indicators: Q and S [21]. Q represents the modularity value, where Q > 0.3 indicates a significant clustering structure. S refers to the silhouette value, which measures the consistency and representativeness of clusters. An S value close to 1 suggests strong cluster cohesion, with S > 0.5 meeting the clustering criteria, and S > 0.7 indicating highly significant clusters [20,21]. Centrality refers to the position of a node that occupies a central location within a network or a keyword or article that connects multiple other articles. Such nodes play a pivotal role as key intermediaries, facilitating connections and interactions within the network. Strength is a quantitative measure that reflects the abrupt increase in intensity. A higher value indicates a more rapid and significant growth rate of the emergent term within a short timeframe. The Log-Likelihood Ratio (LLR) algorithm is a statistical methodology grounded in the principles of log-likelihood ratios, designed to detect and delineate cluster structures within literature networks [20]. As one of the cornerstone algorithms of CiteSpace, LLR is frequently chosen as the optimal clustering model for bibliometric analyses due to its robust performance.

## 3. Results and Discussion

**Figure 1:**
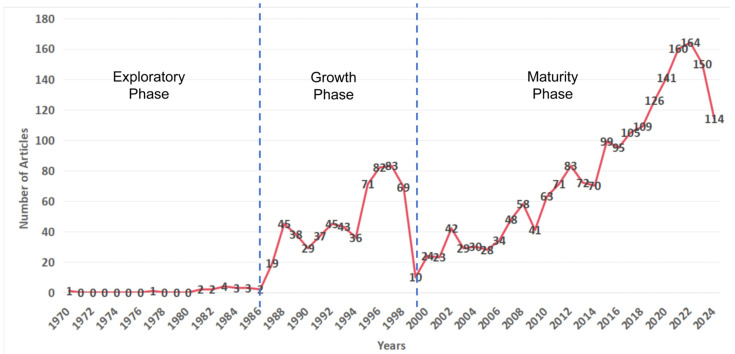
Trends and phases of publications in this field.

A statistical analysis of the annual publication volume reveals that, in combination with the trends in publication volume and the theory of scientific evolution [21], the development of this field can be roughly divided into three distinct phases (Figure 1). From 1970 to 1986, the field was in its exploratory phase, with a total of 17 publications. After the first article was published in 1970, there was a period of silence for 8 years before further related studies began to appear. During this period, the literature primarily focused on the application of observational data in waterbird conservation [25], or on using waterbird count data to study population dynamics of specific species [26,27,28]. From 1987 to 1998, the field entered its growth phase, characterized by a stable increase in annual publication volume. In 1997, the annual publication volume reached its peak for this period. Research during this phase primarily concentrated on using long-term observational data to study the conservation status of waterbirds in protected areas, explore the factors influencing population changes, and predict population trends based on long-term data [29,30,31]. From 1998 to 2024, the field reached its maturity phase, with annual publication volume continuing to increase steadily and reaching new heights. This indicates that, during this period, an increasing number of scholars began to focus on the field and actively contribute to waterbird conservation based on citizen science data [32,33].

### 3.1. Analysis of Research Areas

**Figure 2:**
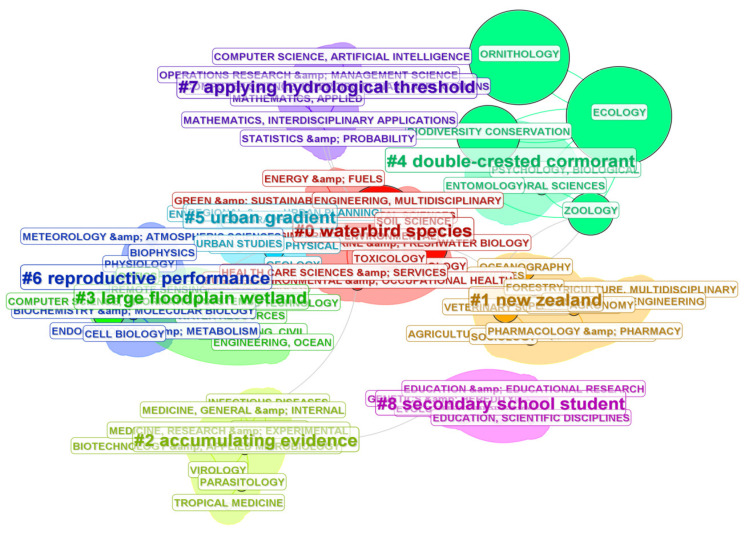
Research Area Clustering Diagram. Nodes of the same color belong to the same cluster. Larger nodes indicate higher publication output within that research area. Connecting lines represent collaborative relationships between different research fields.

The collected publications in this study span 79 research areas. The top ten research areas by publication volume are Ecology, Ornithology, Environmental Sciences, Biodiversity Conservation, Marine and Freshwater Biology, Zoology, Multidisciplinary Sciences, Water Resources, Veterinary Sciences, and Evolutionary Biology. Data on cluster IDs, clusters’ size, clusters’ Silhouette, and clusters’ labels are presented in Table 2. These 79 research areas were clustered into nine major categories, as illustrated in Figure 2, with a summary of the clustering network provided in Table 3. The results indicate that four of the top ten research areas are associated with Cluster #4 Double-crested Cormorant. Research on the Double-crested Cormorant (*Nannopterum auritum*) is predominantly concentrated in the fields of Ecology, Ornithology, Biodiversity Conservation, and Zoology. Additionally, Clusters #0 Waterbird Species and #3 Large Floodplain also encompass research areas with high publication volumes. From a temporal perspective, early studies in this field were largely focused on specific waterbird species, such as the double-crested cormorant. This reflects an initial phase where research aimed to gather foundational knowledge on the population dynamics, habitat use, and conservation challenges of individual species [34,35,36,37]. Over time, research began to concentrate on specific regions, such as New Zealand or Large Floodplain wetlands. With the accumulation of data and the development of the discipline, significant research areas have increasingly shifted toward theoretical topics, such as reproductive performance and the accumulation of evidence. Notably, the #8 Secondary School Student cluster highlights the growing recognition among scholars that waterbird conservation efforts should start by raising public awareness. Enhancing conservation awareness among secondary school students has been identified as an effective approach to fostering long-term conservation efforts [33].

### 3.2. Analysis of Research Hotspots

**Figure 3:**
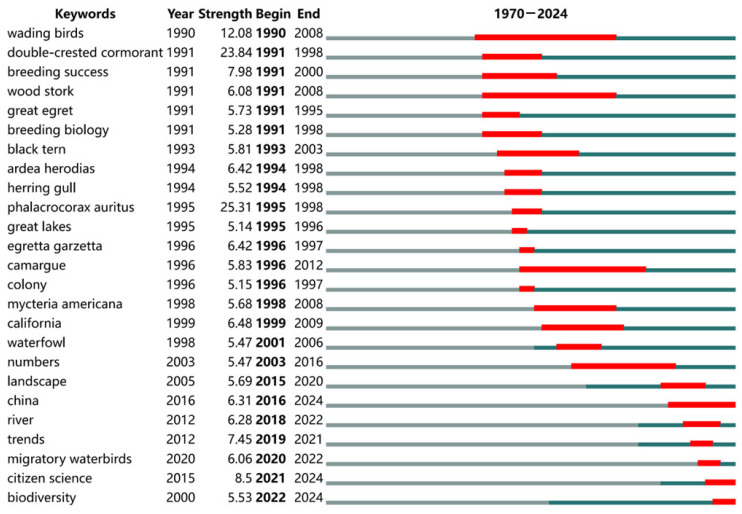
Top 25 Keywords with the Strongest Citations Bursts. The red bars represent the duration of keywords burst periods, while the blue bars indicate the occurrence and persistence of keywords over time. The begin year serves as a crucial criterion for classification and is therefore highlighted in bold.

In this study, 4387 unique keywords were extracted from 2604 papers. Figure 3 presents the top 25 keywords with the strongest citation bursts. Although related studies began in 1970, the earliest burst keywords among these 25 appeared in 1990. Seventeen of these keywords emerged during the second phase of the field’s development, from 1987 to 1998. During this phase, the keywords primarily focused on specific waterbird species, such as ‘Double-crested Cormorant’, ‘Wood stork’, ‘Great egret and Black tern’. Among these, the *Double-crested Cormorant* (*Phalacrocorax auritus*) stood out as a dominant research hotspot due to its exceptionally high burst intensity. Additionally, specialized terms in avian behavior and ecology, such as *breeding success* and *breeding biology*, were prevalent. At the beginning of this phase, *wading birds* emerged as an early burst keyword with high citation intensity. By contrast, *waterfowl*, which appeared at the end of this phase, achieved its peak burst during the third phase (post-2001). However, the burst duration and intensity for *waterfowl* were shorter and weaker than those for *wading birds*, indicating the latter’s sustained significance in waterbird conservation. Furthermore, geographic hotspots such as the *Great Lakes*, *Camargue*, and *California* exhibited strong citation bursts, highlighting their importance as trending research areas. While the burst intensity for *Camargue* was lower than that of *California*, its burst lasted 16 years, emphasizing its long-term significance as a critical site for waterbird conservation.

As the field progressed into the third phase, research priorities shifted significantly. Studies transitioned from species-specific investigations to broader ecological metrics at the population level, such as *numbers*, *trends,* and *biodiversity*. During this phase, *migratory birds* gained attention as a research focus, although its citation burst lasted only two years. Meanwhile, *biodiversity* remained a central topic for over two decades but only exhibited a strong citation burst starting in 2022, which continues to the present. Similarly, *citizen science* emerged as a burst keyword in 2021, and *China* in 2016, both maintaining their prominence. These trends suggest that *biodiversity*, *citizen science*, and *China* will remain pivotal research hotspots in the field of waterbird conservation for the foreseeable future.

### 3.3. Evolution of Research Themes

**Figure 4:**
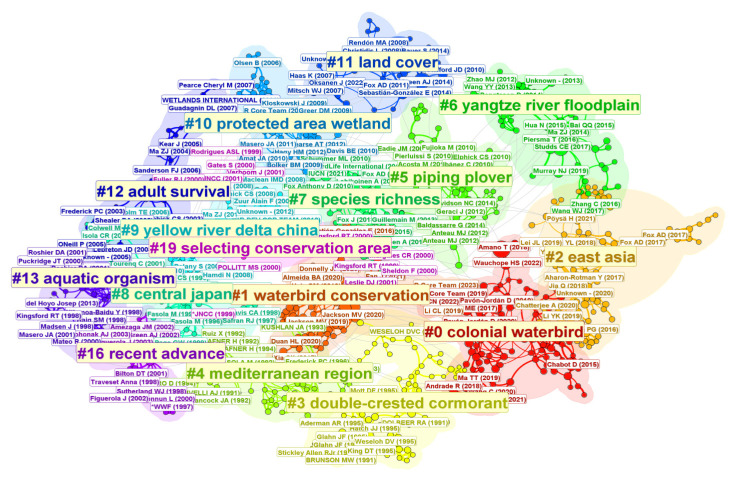
The co-citation network map of the literature. The map contains a total of 1674 nodes and 3685 edges. Each node represents a single paper, while the edges indicate co-citation relationships between the papers. The size of each node reflects the co-citation frequency of the corresponding paper.

In this study, 2604 papers included 74,264 co-cited references, which were clustered using CiteSpace. The clusters were labeled based on the Log-Likelihood Ratio (LLR) algorithm, resulting in a co-citation network map (Figure 4). The map comprises 1674 nodes and 3685 edges. Each node represents a cited reference, while edges indicate co-citation relationships between the references. The size of each node reflects the co-citation frequency.

#### 3.3.1. Analysis of Key Cited Literature

Key cited literature typically introduces innovative theories or methodologies within a research domain or provides comprehensive summaries of the current state of knowledge, guiding future research directions [38]. CiteSpace analysis function identifies authoritative papers with high citation and burst values. Table 4 summarizes the top 10 most-cited papers in this research field.

Amano and Green authored the two most-cited papers. Amano’s article (burst strength = 15.92, 2019–2024) is the most-cited and ranks second in burst strength. It exhibited citation bursts beginning in its second year of publication and has remained influential. This study modeled time-series abundance data for 461 waterbird species across 25,769 survey sites globally, demonstrating that sociopolitical instability leads to biodiversity loss and undermines conservation efforts [39]. Green’s paper holds the highest burst strength (burst strength = 18.85, 2014–2019). It experienced a citation burst from the year of its publication, lasting for six years. This study highlights the critical role of waterbirds as predators, herbivores, and vectors of seeds, invertebrates, and nutrients within aquatic ecosystems. It also explores methods for quantifying the economic value of waterbird ecosystem services through case studies [40].

Several highly cited papers exhibit significant citation bursts shortly after publication, underscoring their pivotal role in shaping the research field. These include Ma (2010) (burst strength = 13.39, 2012–2015) [42], Studds (2017) (burst strength = 11.67, 2020–2022) [41], Bates (2015) (burst strength = 10.47, 2016–2020) [44], Wang (2018) (burst strength = 10.09, 2021–2024) [8], and two papers by Jackson, published in 2020 (burst strength = 9.14, 2021–2024) and 2019 (burst strength = 7.76, 2020–2024) [5,45]. These papers gained substantial citations within 1–3 years of publication, marking them as transformative works in the field.

Ma’s study reviews habitat variables influencing waterbird use of wetlands, emphasizing that wetland management should integrate comprehensive knowledge of the entire ecosystem. It highlights the need to balance spatial and temporal variability with the diverse habitat requirements of different waterbird species, offering management recommendations to enhance habitat quality [42]. Studds and Wang focus on migratory waterbirds, analyzing population trends and habitat changes [8,41]. Studds investigates the population dynamics of shorebirds refueling on the Yellow Sea’s tidal mudflats and explores the impact of stopover habitat changes, concluding that such changes severely constrain migratory populations [41]. Wang assesses population trends of 260 waterbird species in China, identifying habitat loss as a critical threat [8]. Bates contribute methodologically with their work on the *lmer* function in the *R lme4* package for maximum likelihood estimation of linear mixed-effects parameters. This tool rapidly became a cornerstone for wetland ecosystem research [44]. Jackson’s two papers explore waterbird utilization of artificial tidal habitats (2019) and the extent of coastal habitat use along the East Asian-Australasian Flyway (2020) [5,45]. Both emphasize the necessity of conserving and enhancing natural habitats while managing artificial wetlands, advocating for these actions as priorities in waterbird conservation.

#### 3.3.2. Analysis of Thematic Clusters

In the network generated for this study, the Q value is 0.937, demonstrating a highly significant network structure. As shown in Table 5, all 16 clusters have silhouette values exceeding 0.7, with an average silhouette value above 0.9. This indicates that the research themes within each cluster are clearly defined and exhibit a high degree of focus. The thematic clusters can be categorized into five major groups: ecological concepts and indicators, ecological conservation and management, species and taxonomy, geographical regions and ecosystems, and research advancements.

Key ecological concepts and indicators relevant to waterbird conservation include species richness, land cover, and adult survival. Scientists have made extensive use of citizen science data to explore these areas. One of the most notable studies utilized 50 years of citizen science data from 733 non-breeding (particularly wintering) sites for 12 waterbird species to identify habitat requirements and changes in habitat use [47]. This study revealed large-scale shifts in range and distribution driven by climate and environmental changes. Reid applied multivariate statistical analysis (MVA) to 51,000 survey records of 96 waterbird species from the New Atlas of Australian Birds [48]. The study concluded that providing environmental flows could enhance the health of degraded catchment wetlands by supporting the reproduction and population maintenance of colony-nesting waterbirds. Similarly, Monticelli used a six-year capture-mark-recapture dataset for adult breeding terns to model survival rates, demonstrating that both citizen science and survey data are reliable for estimating adult survival rates in terns [49].

To advance waterbird conservation, extensive research has been conducted on ecological conservation and management using citizen science data. Among these studies, *waterbird conservation* has emerged as the most prominent topic. Ma’s work is particularly influential, summarizing conservation measures undertaken in China’s coastal wetlands over recent decades [50]. Through literature reviews and expert surveys, Ma provided recommendations for future conservation actions in three areas: policy and management, habitat conservation and management, and multi-stakeholder participation [50]. Research on *selecting conservation areas* began earlier, and with growing awareness of migratory bird conservation networks, identifying critical areas and optimizing network expansion have become key focuses [52]. Researchers have found that prioritizing complementarity among sites, rather than applying uniform criteria to each site, enhances species representation and maintains robustness over time [50,52].

The *Wetlands Reserve Program* (*WRP*) is a crucial component of conservation networks [63]. The effectiveness of reserve networks is closely tied to animal movement within and beyond protected areas [64]. WRP has the potential to complement reserve networks in inland regions, playing a vital role in reducing human disturbances to migratory waterbirds and safeguarding biodiversity [51].

Prominent species in waterbird conservation research include *colonial waterbirds*, *double-crested cormorants*, *piping plovers*, and *aquatic organisms*, with *colonial waterbirds* forming the largest research cluster. The average publication year for this cluster is 2019, indicating its emergence as a relatively new field. The most influential paper in this cluster, published in 2024, examines the impacts of human activities on shoreline waterbird diversity. It highlights that artificial wetlands can serve as temporary habitats for waterbirds; however, converting shorelines into fishponds and rice paddies reduces the ability of wetlands to sustain waterbird diversity [53]. These findings provide critical insights for designing effective management strategies for waterbirds and wetland habitats in floodplain lakes. Species-specific studies frequently focus on the role of waterbirds in dispersing seeds and aquatic organisms. While waterbirds’ role as dispersers is widely recognized, a comprehensive evaluation of their effectiveness requires systematic field studies and re-analysis of existing species occurrence and dietary datasets. Such efforts could better leverage socio-economic factors to positively influence waterbird conservation [54,55,56].

In terms of geographic regions and ecosystems, research hotspots include *East Asia*, the *Mediterranean region*, the *Yangtze River floodplain*, *Central Japan*, and the *Yellow River Delta*. In 2020, Cao organized a special issue focusing on *East Asia*, which summarized the biogeographic populations of ten key Anatidae species [57]. The issue covered their current and historical abundance and distribution, migratory routes, and critical sites during key phases of their annual cycle [57]. It emphasized that effective analysis requires the active cooperation of biologists and field managers from different countries involved in monitoring, research, and collaborative programs. Among the other four regional keywords, three are situated in *East Asia*. Additionally, the medium-sized clusters in this research demonstrate that geographic regions are central to the field, with the *East Asian–Australasian Flyway* (*EAAF*) standing out as a priority area. These findings underscore the pivotal role of regional studies in advancing waterbird conservation, with a specific focus on the East Asia flyway [58,59,60,61].

## 4. Conclusions

This study provides a systematic analysis of the application of citizen science in waterbird conservation. First, it delineates distinct developmental phases of the discipline, quantifying its progression over time. Second, it employs clustering methods to group research fields, clearly identifying key areas and outlining the developmental trajectory of the discipline. By analyzing research hotspots across different periods, the study also forecasts future trends, offering valuable insights into prospective research directions. Additionally, a comprehensive understanding of the field is achieved through co-citation analysis and an examination of research topic evolution.

While bibliometric methods enable the rapid identification of critical topics and trends, facilitating a swift and comprehensive understanding of the field, this study is limited to analyzing published literature. Newly published papers often have low citation rates, which can lead to time-lag effects in the analysis, as seen in the decline in publication volume towards the end of the third development phase. Furthermore, although the WOS Core Collection includes authoritative articles, it does not encompass all journal publications, potentially introducing biases due to incomplete data retrieval. While the papers selected for this study were identified through keyword searches, it is evident that some do not primarily focus on citizen science as their main research content. According to Dr. Chen’s theory, in bibliometric analysis, it is preferable to include a broader range of papers rather than risk omission. Consequently, although this approach has facilitated significant progress in mapping the development of the field and rapidly identifying key literature, researchers should not solely rely on these advanced methods. Instead, they must also engage in selective reading of the literature. Finally, the reasons behind the decline in annual publication volume at the end of the second development phase remain unclear. It is hoped that, with the continued advancement of the discipline, expansion of search databases, and improvements in analytical techniques, these issues will be addressed in future research.

## Figures and Tables

**Table 1:** The key information of the published literature.

Description	Results
Timespan	1970–2024
Sources (Journals, Books, etc.)	430
Documents	2604
Annual Growth Rate	9.17%
Average citations per doc	19.02
References	75,390
Keywords	4387
Authors	7199
Authors of single-authored docs	232
Co-Authors per Doc	4.57
International co-authorships	23.54%

**Table 2:** Summary of the largest 9 clusters of research areas. “Size” represents the counts of research areas of each Cluster. “Average Year” denotes the mean publication year of the literature within this cluster.

Cluster ID	Size	Silhouette	Label (LLR)	Average Year
0	11	1	Waterbird Species	1997
1	11	1	New Zealand	2002
2	9	0.924	Accumulating Evidence	2012
3	9	1	Large Floodplain	2006
4	7	1	Double-crested Cormorant	1987
5	6	0.976	Urban Gradient	1999
6	6	0.917	Reproductive Performance	2009
7	6	0.984	Applying Hydrological Threshold	2010
8	5	1	Secondary School Student	2011

**Table 3:** Summary of the top 10 research areas by publication volume.

Counts	Centrality	Cluster ID	Research Areas
1187	0.09	4	Ecology
1065	0	4	Ornithology
533	0.82	0	Environmental Science
407	0	4	Biodiversity Conservation
204	0.64	0	Marine and Freshwater Biology
197	0.26	4	Zoology
95	0	3	Multidisciplinary Sciences
86	0.09	3	Water Resources
56	0.09	1	Veterinary Sciences
50	0.13	8	Evolutionary Biology

**Table 4:** Top 10 most-cited papers according to cited counts.

Cited Counts	Cluster ID	Bursts	Representative Papers
48	0	15.92	Amano T, 2018, NATURE, V553, P199, DOI 10.1038/nature25139 [39]
40	11	18.85	Green AJ, 2014, BIOL REV, V89, P105, DOI 10.1111/brv.12045 [40]
28	6	11.67	Studds CE, 2017, NAT COMMUN, V8, P0, DOI 10.1038/ncomms14895 [41]
23	9	13.39	Ma ZJ, 2010, WETLANDS, V30, P15, DOI 10.1007/s13157-009-0001-6 [42]
23	1	0	R Core Team, 2023, R LANG ENV STAT COMP, V0, P0, DOI 10.4135/9781473920446.N12 [43]
22	7	10.47	Bates D, 2015, J STAT SOFTW, V67, P1, DOI 10.18637/jss.v067.i01 [44]
22	1	9.14	Jackson MV, 2020, BIOL CONSERV, V247, P0, DOI 10.1016/j.biocon.2020.108591 [5]
22	1	7.76	Jackson MV, 2019, ECOL EVOL, V9, P2505, DOI 10.1002/ece3.4895 [45]
21	1	10.09	Wang XD, 2018, AVIAN RES, V9, P0, DOI 10.1186/s40657-018-0106-9 [8]
18	7	6.47	IUCN, 2021, THE IUCN RED LIST OF THREATENED SPECIES. 2019-01 (VERSION 6.2), V0, P0, DOI 10.2305/IUCN.UK.2018-2.RLTS.T118264161A163507876.EN [46]

**Table 5:** Table of the thematic clusters. “Size” represents the publication volume under each label. “Silhouette” indicates the clustering degree of each label. “Mean Year” refers to the average publication year of the papers within each label.

Thematic Clusters	Cluster ID	Label (LLR)	Size	Silhouette	Mean Year	Representative Article
Ecological Concepts and Indicators	7	*species richness*	57	0.965	2014	Musilova [47]
	11	*land cover*	44	0.945	2010	Reid [48]
	12	*adult survival*	40	0.986	2005	Monticelli [49]
Ecological Conservation and Management	1	*waterbird conservation*	108	0.971	2019	Ma [50]
	10	*protected area wetland*	46	0.947	2009	Beatty [51]
	19	*selecting conservation area*	20	0.998	1999	Jackson [52]
Species and Taxonomy	0	*colonial waterbird*	117	0.944	2019	Ji [53]
	3	*double-crested cormorant*	81	0.995	1992	Nisbet [54]
	5	*piping plover*	73	0.946	2011	Elphick [55]
	13	*aquatic organism*	38	0.894	1999	Green [56]
Geographical Regions and Ecosystems	2	*East Asia*	93	0.961	2018	Cao [57]
	4	*Mediterranean region*	74	0.991	1993	Hoffmann [58]
	6	*Yangtze river-floodplain*	70	0.978	2014	Wang [59]
	8	*Central Japan*	47	0.967	1998	Maeda [60]
	9	*Yellow River Delta China*	47	0.989	2009	Marquez [61]
Research Advancements	16	*recent advance*	29	0.974	2000	Green [62]

## Data Availability

Data used in this study are available through a search on WOS (https://www.webofscience.com/wos/, accessed on 20 September 2024).
